# Effects of iota-carrageenan on ocular *Chlamydia trachomatis* infection in vitro and in vivo

**DOI:** 10.1007/s10811-018-1435-0

**Published:** 2018-03-13

**Authors:** Aleksandra Inic-Kanada, Elisabeth Stein, Marijana Stojanovic, Nadine Schuerer, Ehsan Ghasemian, Ana Filipovic, Emilija Marinkovic, Dejana Kosanovic, Talin Barisani-Asenbauer

**Affiliations:** 1 OCUVAC – Center of Ocular Inflammation and Infection, Laura Bassi Centres of Expertise, Center for Pathophysiology, Infectiology and Immunology, Medical University of Vienna, Kinderspitalgasse 15, 1090 Vienna, Austria; 2 Department of Research and Development, Institute of Virology, Vaccines and Sera – TORLAK, Belgrade, Serbia

**Keywords:** Carrageenan, Trachoma, Natural products, Chlamydia

## Abstract

Ocular chlamydial infections with the ocular serovars A, B, Ba, and C of *Chlamydia trachomatis* represent the world’s leading cause of infectious blindness. Carrageenans are naturally occurring, sulfated polysaccharides generally considered safe for food and topical applications. Carrageenans can inhibit infection caused by a variety of viruses and bacteria. To investigate whether *iota*-carrageenan (I-C) isolated from the red alga *Chondrus crispus* could prevent ocular chlamydial infection, we assessed if targeted treatment of the conjunctival mucosa with I-C affects chlamydial attachment, entry, and replication in the host cell. Immortalized human conjunctival epithelial cells were treated with I-C prior to *C*. *trachomatis* infection and analyzed by flow cytometry and immunofluorescence microscopy. In vivo effects were evaluated in an ocular guinea pig inclusion conjunctivitis model. Ocular pathology was graded daily, and chlamydial clearance was investigated. Our study showed that I-C reduces the infectivity of *C*. *trachomatis* in vitro. In vivo results showed a slight reduced ocular pathology and significantly less shedding of infectious elementary bodies by infected animals. Our results indicate that I-C could be a promising agent to reduce the transmission of ocular chlamydial infection and opens perspectives to develop prophylactic approaches to block *C*. *trachomatis* entry into the host cell.

## Introduction

Ocular (Lietman et al. 1998) and genital (Norman 2002) chlamydial infections are common worldwide (146 million cases/year) (ECDC 2015). Repeated infections with *Chlamydia trachomatis* ocular serovars A, B, Ba, and C trigger fibrotic processes in the affected ocular tissues leading to trichiasis, corneal opacity, and in further progression to complete loss of sight (Mariotti et al. 2009). Apart from reducing the quality of life, ocular chlamydial infections can lead to a sight-threatening complication called trachoma. Trachoma is considered a neglected tropical disease and is the world’s leading infectious cause of preventable blindness (Taylor et al. 2014). Currently, control in the endemic populations is achieved through mass drug administration (Frick et al. 2001) and implementation of the SAFE strategy (*S* stands for “surgery” for trachomatous trichiasis, *A* stands for “antibiotic” treatment, *F* stands for “facial” cleanliness, and *E* stands for “environmental” improvement such as general community hygiene, adequate water supply, and construction of sanitary facilities) (WHO 2014).

To more efficiently combat the spread of *Ct* in these rural poverty areas, novel methods for treatment and prevention of *C*. *trachomatis* infection are needed. There exist various studies exploring the potential of natural products for developing new anti-chlamydial treatment modalities (Brown et al. 2016). Polyphenolic (Daglia 2012), lipidic (Bergsson et al. 1998), proteinaceous compounds (Yasin et al. 1996), and polyherbal formulations have demonstrated significant anti-chlamydial activity (Talwar et al. 1995). Overall, natural products show significant potential in treating chlamydial infections and the development of novel drugs based on natural products may help in the global management of *Chlamydiae*-related infections.

Carrageenans are naturally occurring anionic sulfated polysaccharides (SPs), which are present in a number of seaweeds of the class Rhodophyceae, such as *Chondrus*, *Gigartina*, *Hypnea*, and *Eucheuma* (Lahaye 2001; Necas and Bartosikova 2013). They have an excellent and well-documented safety profile for long-term use, since they have been widely used in the food, pharmaceutical, and cosmetic industry as additives, thickeners, and emulsifiers. The Food and Drug Administration has listed carrageenan compounds as “generally recognized as safe” (GRAS) in 1973 (FDA SCOGS (Select Committee on GRAS Substances) n.d.). Carrageenans consist of alternate units of D-galactose and 3,6-anhydro-galactose, both sulfated and nonsulfated, joined by α-1,3 and β-1,4-glycosidic linkage (Necas and Bartosikova 2013). Depending on the allocation of the sulfate groups on the main structures, carrageenan is classified into various types (λ, κ, ι, ε, μ), all containing 22 to 35% sulfate groups, of which *λ*, *κ*-, and *ι*-carrageenans are widely used in the food industry (Vera et al. 2011; Necas and Bartosikova 2013).

Different types of carrageenans have been found to be active against a variety of viruses, including human papillomavirus (HPV) (Buck et al. 2006; Roberts et al. 2007; Levendosky et al. 2015), herpes simplex virus (Carlucci et al. 1999), rhinovirus (Grassauer et al. 2008), influenza (Leibbrandt et al. 2010), metapneumovirus (Klimyte et al. 2016), and rabies (Luo et al. 2015). Antiviral potential against human immunodeficiency virus was already described two decades ago (Gonzalez et al. 1987) and has been reviewed recently (Damonte et al. 2004). Although it has been proven that carrageenans show strong antiviral properties, the exact mechanisms of action and structural determinants for these compounds are not fully elucidated. It has been hypothesized that carrageenans exert their antiviral activity by direct interaction with virus particles at an early stage of viral infection (Gonzalez et al. 1987). Initial attachment of the virus to human cells is mediated by the interactions between the virion and a type of cell surface glycosaminoglycan (GAG) heparan sulfate (Buck et al. 2006). Carrageenans closely resemble heparan sulfate and could interact directly with the viral particles, preventing attachment to the respective receptors on the cell surface. It has also been shown that *iota*-carrageenan (I-C) possesses antiviral activity not only due to direct interaction with influenza A virus but also due to coating of cellular structures, hindering receptor binding sites (Buck et al. 2006; Leibbrandt et al. 2010).

The effects of carrageenans also have been described in the context of bacterial infections. The antimicrobial action of I-C on food-borne pathogenic bacteria has been described and shown that the inhibitory effect of carrageenans was not bactericidal but bacteriostatic (Yamashita 2001). Moreover, the removal of sulfate residues eliminated the bacteriostatic effect of I-C, suggesting that the sulfate residue(s) in carrageenan play an essential role in this mechanism.

Studies investigating the effects of SPs to block chlamydial infection are sparse. It has been shown that exogenous heparin and heparan sulfate inhibit chlamydial infection (Zhang and Stephens 1992). Although this study used *C*. *trachomatis* lymphogranuloma venereum serovar L2 as their model organism, their results might be translatable to ocular strains since it was shown that for both serovariants heparan sulfate-like-mediated interactions between *C*. *trachomatis* and eukaryotic cells are critical mediators of infectivity (Chen and Stephens 1994). Moreover, the ability of different SPs to inhibit the infection of cervix derived human epithelial cells ME180 with *C*. *trachomatis* serovar E was studied and included different types of carrageenan and GAGs (e.g., heparin, heparan sulfate, and hyaluronic acid). This in vitro study was important as it demonstrated that I-C and other types of SPs block infection of ME180 with *C*. *trachomatis* serovar E by preventing bacterial attachment to the host cell (Zaretzky et al. 1995). This is in line with numerous reports showing that the entry of *Chlamydia* into the host cell is dependent on elementary bodies (EBs) interactions with GAGs exposed on the surface of the host cells (Chen and Stephens 1994; Moelleken and Hegemann 2008).

It is well known that a successful prevention of an infection depends on how efficient a certain product/drug is in preventing the entry of the infectious agent into the host organism. For chlamydial infections, this would mean inhibiting/preventing the infection of ocular and genital mucosal epithelium. Most importantly, even before taking any product/drug into consideration for treatment of chlamydial infections, their safety profiles on mucosal surfaces must be established. Recently, it has been shown that carrageenans, including I-C, were not cytotoxic and did not induce proinflammatory cytokines in epithelial cell lines HT-29 and HCT-8 (McKim Jr. et al. 2016). Moreover, in studies investigating possibilities of using carrageenans as microbicides, it has been shown that a topical application of carrageenan is safe for mucosal epithelium (Coggins et al. 2000; Grassauer et al. 2008; Eccles et al. 2015).

In the present study we used (i) an in vitro infection model highly resembling the human ocular surface: immortalized human conjunctival epithelial (HCjE) cells infected with *C*. *trachomatis* ocular serovar B (CtB) (Stein et al. 2013; Rahn et al. 2016) and (ii) an in vivo guinea pig inclusion conjunctivitis infection model, which uses *Chlamydia caviae* (GPIC), a natural pathogen of the guinea pigs, as the infectious agent (Rank and Whittum-Hudson 1994; Belij-Rammerstorfer et al. 2016; Filipovic et al. 2017) to investigate if targeted treatment of the ocular conjunctival mucosa with I-C derived from *Chondrus crispus* can block chlamydial attachment, entry, and by these means also bacterial replication in the ocular epithelial cells.

## Material and methods

### Chlamydial strains

*Chlamydia trachomatis* ocular serovar B (CtB) (ATCC VR-573) and *Chlamydia caviae* (GPIC) (kindly provided by Prof. Roger G. Rank) were propagated in McCoy cells (ATCC CRL-1696) according to standard procedures (Caldwell et al. 1981). Harvested stocks were centrifuged at 200 × *g* to pellet cellular debris, resuspended in sucrose-phosphate-glutamate buffer (SPG) containing 0.01 M sodium phosphate (pH 7.2), 0.25 M sucrose, and 5 mM L-glutamic acid.

### Cell culture

HCjE cells, kindly provided by Prof. Ilene Gipson (Schepens Eye Research Institute, Harvard Medical School, Boston), were maintained in keratinocyte serum-free medium supplemented with bovine pituitary extract, 0.2 ng mL^−1^ EGF and 1% penicillin/streptomycin (Life Technologies, UK) at 37 °C/5% CO_2_ and 95% humidity. The medium was changed every second day, and cells were passaged at 70% confluence.

### In vitro infection assay

For in vitro infection assays, HCjE cells were maintained in keratinocyte serum-free medium (Life Technologies, UK) at 37 °C/5% CO_2_ and 95% humidity. The medium was changed every second day, and the cells were passaged at 70% confluence. Cells were harvested by trypsinization (0.05% Trypsin/0.02% EDTA in PBS, GE Healthcare) and seeded at a density of 150.000 cells per well in 24-well plates (Greiner Bio-One, Austria). An aqueous solution containing 2.4 mg mL^−1^ I-C (0.24% *w*/*v*; I-C derived from *Chondrus crispus*; FMC Biopolymers, USA) and 36.4 mg mL^−1^ mannitol (Sigma Aldrich, Germany) was formulated to test the antimicrobial activity against CtB. The dose was chosen according to the results of a preliminary experiment—2.4 mg mL^−1^, which was the concentration of an obtained liquid solution, was the most efficient dose in inhibiting chlamydial infection (serial dilutions 2.4, 1.2, 0.6, and 0.3 mg mL^−1^) showed lower dose-dependent inhibiting effect and all doses did not exhibit any cytotoxicity on HCjE cells. The I-C solution was filtered through a 0.22-μM (Sarstedt, Germany) sterile filter and stored at 4 °C. The unbuffered I-C solution had a pH between 6.8 and 7.4 and an osmolarity between 210 and 220 mOsm kg^−1^. A sterile aqueous solution containing 36.4 mg mL^−1^ mannitol served as a placebo control.

Confluent cultures of HCjE cells were treated with I-C solution in the given concentration and subsequently infected with 1 × 10^4^ inclusion-forming unit (IFU) CtB, resuspended in SPG, per well. Placebo-treated cells and cells without any pre-treatment served as controls. A second group of mock-infected cells, treated only with either I-C or placebo, were assessed in the same manner to evaluate possible cytopathic effects of the formulations by phase-contrast microscopy. HCjE cells were incubated for 2 h at 37 °C to ensure CtB attachment. After 2 h, the medium was changed to standard growth medium without antibiotics and cells were incubated for 48 h at 37 °C/5% CO_2_ and 95% humidity. Cells were fixed with ice-cold methanol for 20 min at − 20 °C, air-dried, and stained with an anti-Chlamydia LPS-FITC labeled antibody (Clone B410F, Pierce Biotechnology, USA) diluted 1:20 in PBS for 30 min at 37 °C. CtB IFUs were counted on a Zeiss AxioObserver microscope using Tissue FaxSi Software (Tissuegnostics, Austria) for acquisition. HCjE cells were counterstained with DAPI. Inclusion size was measured in 20 high power fields per sample. All experiments were repeated three times and were performed in triplicates.

### Adhesion assay

To assess changes in the efficiency of attachment of CtB elementary bodies (EBs) to the cell surface of I-C-treated HCjE cells, a flow cytometric adhesion assay with carboxyfluorescein succinimidyl ester (CFSE) labeled CtB EBs was performed according to a previous study (Molleken et al. 2010). Briefly, CtB EBs resuspended in SPG were labeled with 20 μmol L^−1^ CSFE (ebioscience, Vienna Austria) for 90 min at room temperature (RT) as previously described (Schnitger et al. 2007). EBs were washed three times with PBS containing 1% BSA to remove excess CSFE. Confluent monolayers of HCjE cells (150.000 cells per well) were treated with 2.4 mg mL^−1^ I-C or placebo solution for 5 min at 37 °C/5% CO_2_ and 95% humidity. After 5 min of incubation of HCjE cells with I-C, labeled CtB EBs at a MOI (multiplicity of infection) of 10 were added into wells and further incubated for 1 h at 37 °C/ 5% CO_2_ and 95% humidity. Cells were then washed with PBS, harvested by trypsinization, and fixed with 2% paraformaldehyde in PBS for 15 min at RT. Fluorescence of cells infected with CFSE-labeled CtB was assessed with a FACS Calibur flow cytometer (BD Biosciences, Germany). The experiment was repeated twice and both times was performed in triplicates.

### In vivo infection

All animal experiments were approved by the Torlak Institute and conformed to the Serbian laws and European regulations on animal welfare (Approval No. 323-07-01577/2016-05/12). All animals were handled in strict accordance with good animal practice as defined by the Serbian code of practice (published in Sluzbeni Glasnik No. 41/9) for the care and use of animals for scientific purposes, the Guide for the Care and Use of Laboratory Animals of the Torlak Institute (2133/1, 21. 04. 2011), a Basel declaration that is committed to the 3R principle (replace, reduce, refine). Every effort was made to minimize animal suffering. Any animals found to be requiring treatment were given appropriate veterinary care. We did not observe any unexpected deaths of animals during this study.

In vivo efficacy of I-C treatment was evaluated on guinea pigs Hartley Strain (300–350 g) anesthetized with a mixture of ketamine (30 mg kg^−1^) and xylazine (2 mg kg^−1^) applied intramuscularly prior any manipulation. Two sets of in vivo experiments were done. In the first experiment, we evaluated the impact of local I-C application prior GPIC inoculation on the course of chlamydial infection (pre-treatment). Guinea pigs were treated either with 25 μL per eye of I-C (2.4 mg mL^−1^) or with appropriate placebo solution 2 h before the infection with 1 × 10^4^ IFU of GPIC. In the second experiment, we evaluated the impact of local I-C application during the acute phase of infection (treatment). Guinea pigs infected with 1 × 10^4^ IFU of GPIC were treated for 7 days, daily, either with 25 μL per eye of I-C (2.4 mg mL^−1^) or with appropriate placebo solution starting from day 3 post-infection. The day of GPIC inoculation was considered as day 0 in both experiments. An experienced ophthalmologist who was blinded to the experimental groups observed and scored daily the eyes of guinea pigs in a protection assay (Rank et al. 1995). In brief, the palpebral and the bulbar conjunctivae were evaluated for erythema, edema, and exudation in each animal. Each observation was classified into five categories: (0.5) trace pathologic response, (1) slight erythema or edema of either the palpebral or the bulbar conjunctiva, (2) definite erythema or edema of either the palpebral or the bulbar conjunctiva, (3) definite erythema or edema of both the palpebral and the bulbar conjunctivas, or (4) definite erythema or edema of both the palpebral and the bulbar conjunctivas plus the presence of exudate.

### Determination of GPIC IFUs from conjunctival swabs

For the quantification of GPIC EBs, conjunctival swab samples were collected from guinea pigs while under ketamine/xylazine anesthesia before and 4, 7, 14, and 21 days post-infection. Darcon swabs were used to swab the palpebral and the bulbar conjunctiva and placed in Copan Universal Transport Medium (UTM-RT) System (Copan, Italy). IFUs were determined by inoculation of the obtained swab material onto confluent cultures of McCoy cells (ATCC CRL-1696). Centrifugation at 200 × *g* for 1 h was carried out to ensure attachment of EBs. After incubation for 24 h at 37 °C/5% CO_2_ and 95% humidity in the presence of 1 mg mL^−1^ cyclohexamide (Sigma Aldrich) cells were fixed in ice-cold methanol and stained with a FITC-conjugated monoclonal antibody against Chlamydia LPS (1:20 in PBS, Clone B410F, Pierce Biotechnology, USA). IFUs were counted using a epifluorescence microscope (Zeiss AxioObserver, Zeiss, Germany).

### Statistical analysis

To assess statistically significant differences between the treatment groups, a two-way ANOVA followed by Bonferroni multiple comparisons test was used. The level of significance was set at *P* < 0.05. All statistical analyses were performed by the software: IBM SPSS Statistics 20.

## Results

### I-C reduced the number of detectable inclusions in vitro

The analyses of HCjE cells infected in vitro with CtB without any pre-treatment or upon I-C and placebo treatment encompassed the evaluation of CtB attachment, the counting of CtB IFUs, and the evaluation of inclusions’ size.

**Fig. 1:**
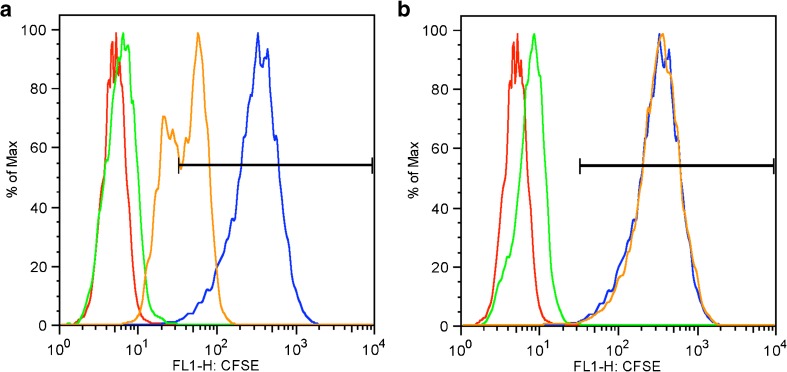
Flow cytometric analyses of HCjE cells infected in vitro with CFSE-labeled CtB in the presence of I-C (**a**) or placebo (**b**) solution. Three independent samples per treatment were analyzed and representative histograms are presented. Each sample consisted of 1 × 10^6^ HCjE cells exposed to CtB at MOI of 10. CtB-infected HCjE, which were not exposed to either I-C or placebo (blue line), non-infected HCjE cells (red line) and non-infected HCjE incubated either with I-C or with placebo solution (green line) were controls. All control cells were incubated under the same conditions (1 h at 37 °C/5% CO_2_ and 95% humidity) and treated in the same manner as HCjE cells infected with CtB in the presence of I-C or placebo (orange line). The gate containing CFSE+ cells is indicated on the histograms (black line)

**Fig. 2:**
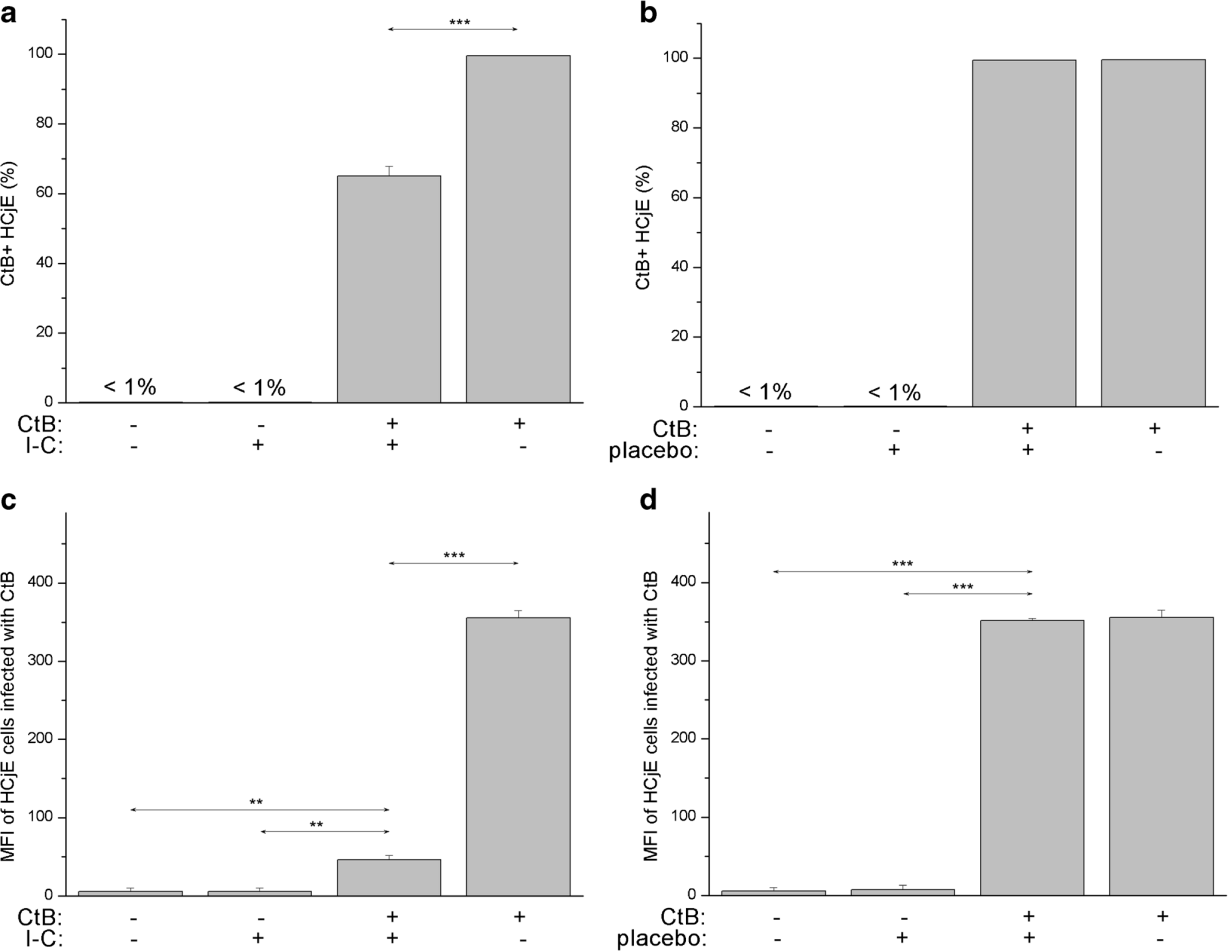
Percentage of CtB+ HCjE cells within the total population of HCjE infected in vitro with CFSE-labeled CtB in the presence of I-C (**a**) and placebo (**b**) solution and the MFI of CFSE signal of HCjE cells exposed to treatments indicated below the plots (**c**, **d**). The gating of CFSE+ HCjE cells is indicated in Fig. 1 Three independent samples per treatment were analyzed and results are presented as a mean value ± SE. Statistical significance between specific treatments is determined by one-way ANOVA followed by Bonferroni test (* *p* < 0.05, ** *p* < 0.005, *** *p* < 0.001). Compared groups are indicated by arrows

Flow cytometric analyses of HCjE cells infected with CFSE-labeled CtB EBs revealed that I-C inhibits CtB attachment to the HCjE cells (Fig. 1). HCjE cells infected with CFSE-labeled CtB, either without pre-treatment or upon placebo treatment were CtB positive (CtB+) (99.4 ± 0.1% and 99.6 ± 0.1%) (Fig. 2a, b). Analysis of viable HCjE cells exposed to CFSE-labeled CtB in the presence of I-C revealed that I-C significantly impaired CtB attachment to HCjE cells because 65.1 ± 2.8% of cells were CtB+ (Fig. 2a; *P* < 0.001 compared either to HCjE cells infected with CFSE-labeled CtB without any pre-treatment or upon placebo treatment). Besides, I-C treatment resulted in a significant reduction of the CFSE signal: the mean fluorescence intensity (MFI) of CtB+ HCjE cells pre-treated with I-C was 59.23 ± 1.34. MFI of HCjE cells pre-treated with I-C was significantly lower in comparison with MFIs recorded for HCjE cells treated either with placebo (351.67 ± 2.85, *P* < 0.001) or without any treatment (355.33 ± 9.17, *P* < 0.001) (Fig. 2c, d).

**Fig. 3:**
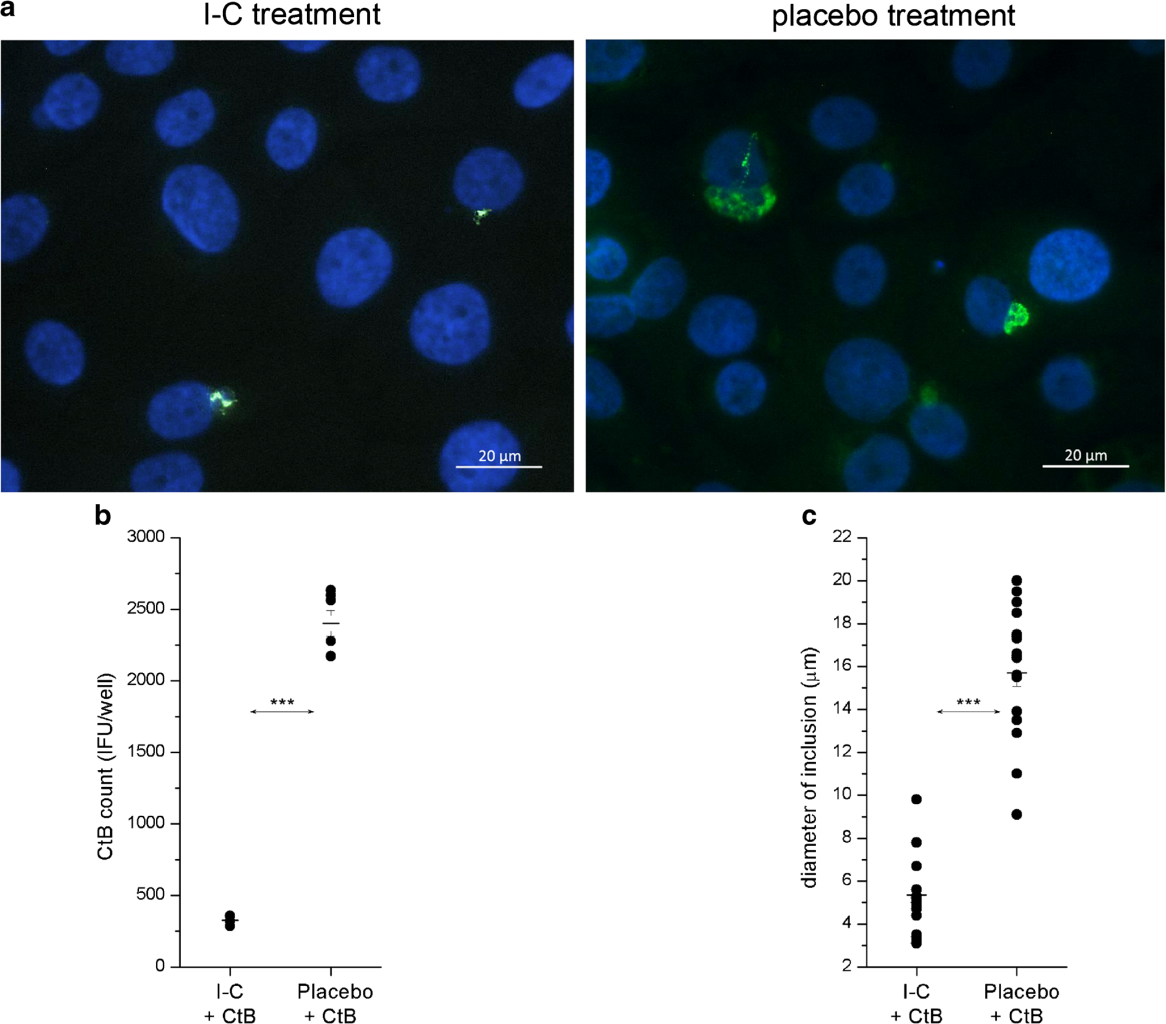
Microscopic analysis of HCjE cells infected with CtB in the presence of I-C and placebo solution (**a**). HCjE cells were seeded at a density of 150.000 cells/well, infected with 1 × 10^4^ IFU of CtB, incubated in appropriate medium supplemented with I-C or placebo for 48 h and then collected for analyses. Representative images are shown. The number (**b**) and the size (**c**) of CtB inclusions are determined. CtB inclusions are identified using FITC-labeled anti-Chlamydia LPS antibody (green dots). HCjE cells were counterstained with DAPI (blue). The significance of the recorded differences is determined by *t* test for independent groups (***P < 0.001)

The counting of CtB IFUs in HCjE cells exposed to specific treatments prior in vitro CtB infection revealed significantly (*P* < 0.001) lower numbers of CtB IFUs for I-C-treated HCjE cells (326 ± 11 IFU well^−1^) in comparison with CtB-infected placebo-treated HCjE cells (2403 ± 89 IFU/well) (Fig. 3a, b). Moreover, inclusions observed upon in vitro CtB infection in I-C-treated HCjE cells (5.35 ± 0.38 μm) were significantly smaller (*P* < 0.001) in comparison to those in placebo-treated HCjE (15.72 ± 0.63 μm; Fig. 3a, c).

### The I-C-treated guinea pigs showed less severe symptoms of ocular chlamydial infection in vivo

Preliminary performed experiments showed that topical treatment with I-C in guinea pigs was well tolerated, as any signs of irritation on the ocular surface were not marked during daily follow-ups.

**Fig. 4:**
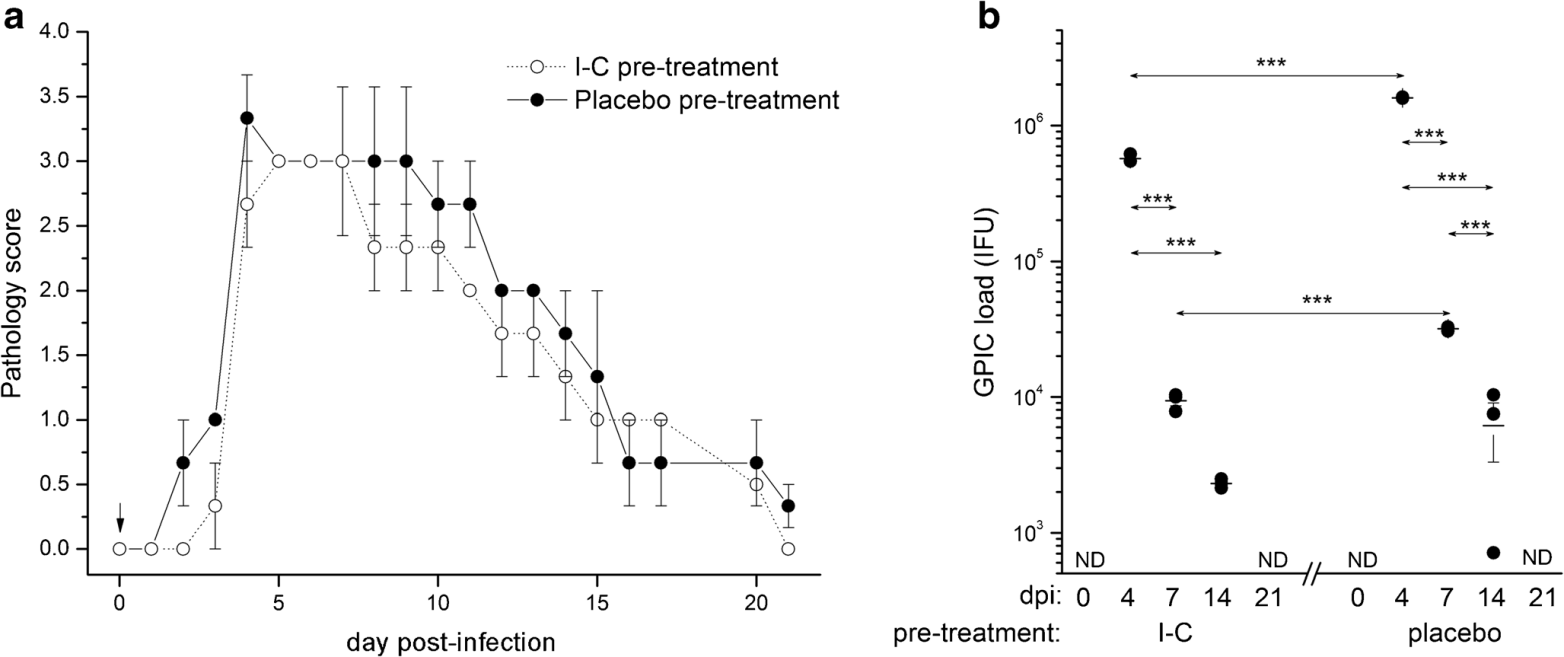
Ocular pathology scores (**a**) and *C. caviae* load (**b**) in guinea pigs infected with a single ocular instillation of *C. caviae* (1 × 10^4^ IFU) 2 h after I-C or placebo treatment. The start of infection is considered as day 0 and the timing of I-C or placebo pre-treatment is indicated by an arrow. The intensity of infection-induced inflammation is scored daily, while *C. caviae* load is determined at previously selected control time-points (days 0, 4, 7, 14, and 21 post-infection). Statistical significance of the observed differences was evaluated using the two-way ANOVA test followed by Bonferroni test (compared groups indicated by arrows; *P < 0.05, **P < 0.005, ***P < 0.001)

In comparison with placebo treatment, a single local I-C administration before GPIC ocular infection did not significantly alter the infection course. During the post-infection period, pathology scores were slightly lower in the I-C pre-treated group compared to the animals that received placebo treatment, although statistically significant differences in intensity of pathology were not marked at any time-point (Fig. 4a). Furthermore, the peak of infection was reached on day 4 post-infection in both groups. The I-C-treated group showed a slightly delayed onset of pathology and less severe symptoms of infection up to day 16. By reaching day 21, all I-C-treated guinea pigs showed no more signs of any pathological events, whereas traces of inflammation were seen within the placebo-treated group. Analysis of changes in chlamydial load during the post-infection period (Fig. 4b) revealed significantly lower absolute numbers of GPIC EBs in I-C-treated guinea pigs on day 4 (*P* < 0.001) and on day 7 post-infection (*P* < 0.001) compared to the corresponding placebo-treated animals. On day 14 post-infection, the number of GPIC EBs was also lower, but not statistically significant, in I-C-treated guinea pigs (*P* > 0.05). No GPIC IFUs could be detected in both groups by day 21 indicating complete clearance of chlamydial infection.

**Fig. 5:**
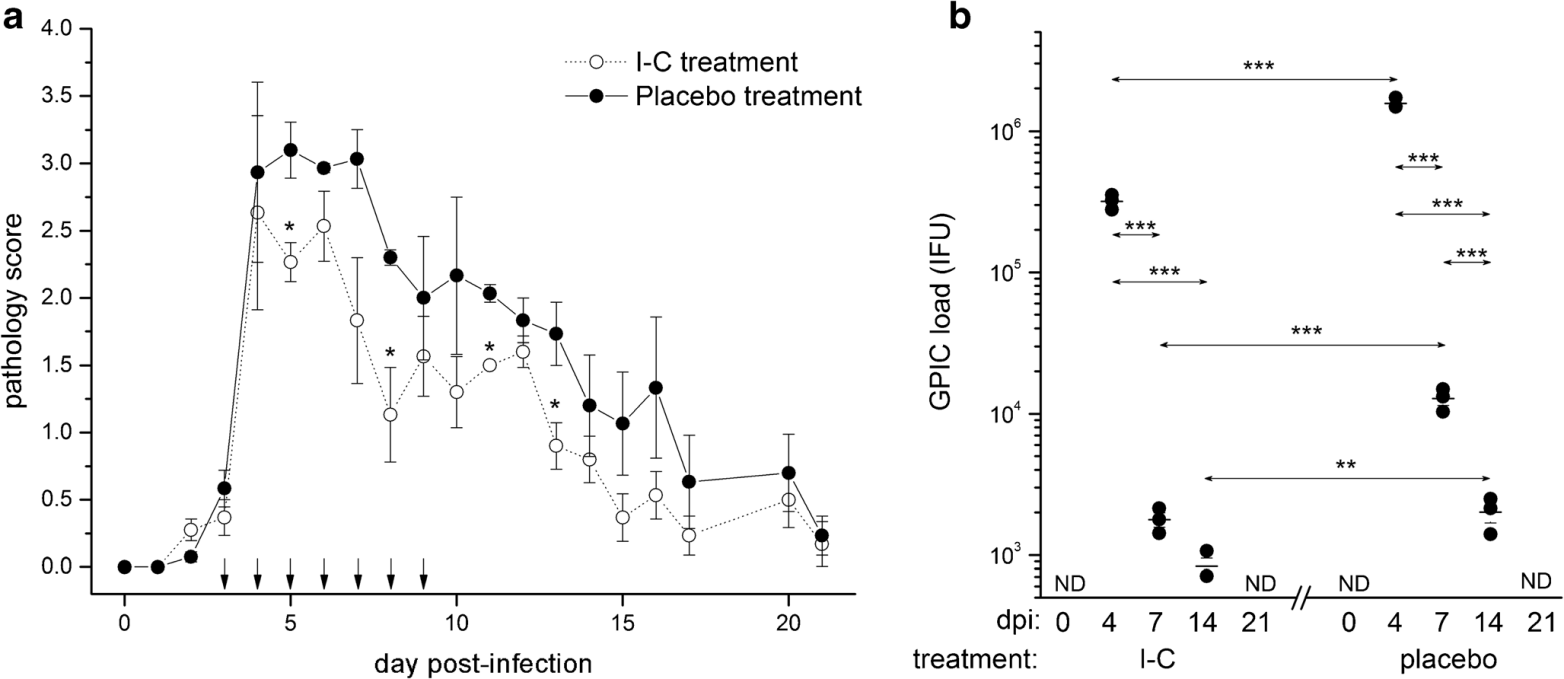
Ocular pathology scores (**a**) and *C. caviae* load (**b**) in guinea pigs infected with a single ocular instillation of *C. caviae* (1 × 10^4^ IFU) as well as guinea pigs treated during the acute phase of infection with I-C or placebo solution. The start of infection is considered as day 0 and the timing of I-C or placebo treatments (from day 3 to day 9 post-infection) are indicated with arrows. The intensity of infection-induced inflammation is scored daily, while *C. caviae* load is determined at previously selected control time-points (days 0, 4, 7, 14, and 21 post-infection). Statistical significance of the observed differences was evaluated using the two-way ANOVA test followed by Bonferroni test (compared groups indicated by arrows; *P < 0.05, **P < 0.005, and ***P < 0.001)

Multiple local I-C applications during the acute phase of ocular GPIC infection exerted more pronounced impact on infection intensity and pathologic response (Fig. 5). A significant reduction in GPIC infectious load due to I-C treatment was accompanied by the reduction in severity of GPIC-induced local inflammation. Pathology scores were lower (*P <* 0.05 on days 5, 8, 11, and 13 post-infection) in the I-C pre-treated group in comparison with the animals receiving placebo treatment (Fig. 5a). The I-C treatment did not shorten the period needed for complete clearance of the infection. Analysis of changes in chlamydial load during the post-infection period revealed significantly lower absolute numbers of GPIC EBs in I-C-treated guinea pigs on day 4 (*P* < 0.001), day 7 (*P* < 0.001), and day 14 (*P* < 0.005) post-infection in comparison with the corresponding placebo-treated animals (Fig. 5b).

## Discussion

The results of the presented study show that I-C effectively reduces CtB infectivity by blocking chlamydial attachment to the epithelial host cells and might be a promising and affordable therapeutic agent against ocular chlamydial infections, especially for endemic trachoma areas.

The effects of a specific I-C treatment on the ocular chlamydial infection were assessed in vitro in HCjE cells and in vivo by using a model of inclusion conjunctivitis in guinea pigs. HCjE cells are considered a valuable and relevant model system for various kinds of ocular surface-targeted research. These cells are derived from healthy human conjunctiva and immortalized by abrogation of p16 control and p53 function before immortalization by expression of human telomerase reverse transcriptase (Gipson et al. 2003). Compared to cervical ME180 cells, which were originally isolated from a highly invasive cervical squamous cell carcinoma, HCjE cells are a more convenient system for testing due to their functional characteristics including similar mucin gene expression as native conjunctival cells (Gipson et al. 2003). An additional limitation of the study on ME180 cells was that they were reported to contain HPV DNA with greater homology to HPV-68 than HPV-18, which might speak against using these cells in infection experiments. Furthermore, the guinea pig model of inclusion conjunctivitis caused by GPIC we used in this study is an important animal model in the field of ocular chlamydial infection as the structural and functional organization of lymphoid tissue underlining the conjunctiva of guinea pigs highly resembles the one of the human ocular region (Rank 2007). This is the main advantage of a guinea pig inclusion conjunctivitis model over the other models of ocular infection of rodents.

Our results show that I-C is able to reduce *C*. *trachomatis* infection in HCjE cells, which is in line with the effect of I-C seen for *C*. *trachomatis* serovar E in genital ME180 cells (Zaretzky et al. 1995). The reduced infectivity of the *C*. *trachomatis* EBs in I-C-treated cells might be explained by the obstruction of their attachment to the I-C-treated host cells. It has been proposed that the inhibitory effect of SPs against *C*. *trachomatis* infection is non-specific and mediated by the strong negative charge of the SPs resulting in prevention of adherence of *C*. *trachomatis* to the host cell by charge repulsion (Zaretzky et al. 1995). The negative influence of I-C on EB attachment to the host cell could further explain the lower number of chlamydial IFUs and/or the smaller size of inclusions upon I-C treatment of host cells we found in our study. Our finding that inclusions in I-C-treated cells are smaller compared to untreated CtB-infected cells suggests that there might be additional mechanisms involved that contribute to the antimicrobial effect of I-C.

Bacterial attachment to mucosal epithelial surfaces is the first step in the establishment of an infection and the specificity of the pathogen–host cell interaction is determined by bacterial surface proteins (adhesins) and their receptors on the host cell surface. GAG structures on the mammalian cell surface play an important role in interactions with many microbial pathogens and are recognized also by *Chlamydia* (Zhang and Stephens 1992; Chen and Stephens 1994; Moelleken and Hegemann 2008). It has also been demonstrated that a chlamydial adhesin, the outer membrane protein B (OmcB), from *C*. *trachomatis* as well as from GPIC, interacts with GAGs on the epithelial host cell surface (Stephens et al. 2001; Fadel and Eley 2007; Moelleken and Hegemann 2008). Interactions of carrageenans with host cell GAGs have been investigated and showed reduced sulfatase activity and redistribution of the cellular GAGs on the host cell surface with potential consequences for cell structure and function (Yang et al. 2012). Considering these findings, it can be hypothesized that I-C in our experiment competitively inhibits the interaction with GAGs on the epithelial cell surface or modulate their availability, which leads to reduced attachment and subsequent infection of the host cells.

Antiviral effects of I-C were investigated in animal models (Fernandez-Romero et al. 2012). It has been shown that I-C could effectively block HPV infection of HeLa and HaCat cells by inhibiting the interaction of the viral capsid with heparin sulfate proteoglycans at the host cell surface as well as that I-C and *kappa*-carrageenan are more efficient in blocking HPV infection than other types of carrageenan (Buck et al. 2006). Differential efficiency of carrageenan to block HPV infection has also been reported showing a protective effect against HPV18 and HPV31 infections, but not against HPV16 and HPV45 (Cruz and Meyers 2013). The efficacy of carrageenan inhibition of dengue virus infection varied depending on the serotype tested (Talarico and Damonte 2016). Strain-specific efficacy of carrageenans depends on their properties but also on the characteristics of the infectious agent. It has been shown that the degree of sulfation has a major impact on the antiviral activity of SPs and that a specific positioning of sulfates might be important for the antiviral activity (Ghosh et al. 2009).

The in vitro obtained data on SPs as anti-chlamydial substances are in line with the strain-specific antiviral activity of SPs. It is demonstrated that the efficacy of SPs, including carrageenans, in inhibition of in vitro chlamydial infection depends on the *Chlamydia* serovar used (Zaretzky et al. 1995; Taraktchoglou et al. 2001).

Despite the existence of data implying a potential beneficial impact of SPs on the course of chlamydial infection, there is no clear evidence of a protective effect of SPs against chlamydial infection in vivo*.* Burillo et al. analyzed the protective capacity of SPs against chlamydial infections in a genital mouse model and did not find any protective effect (Burillo et al. 1998). Our results imply that topical I-C treatment could exert beneficial effects against ocular chlamydial infection but variation in dosage or application schedule must be carefully considered. Our results also imply that prolonged I-C application is required for a beneficial effect. Single I-C application 2 h before chlamydial infection has not resulted in a significant reduction in pathology intensity in comparison with placebo-treated animals. On the other hand, its daily application resulted in a significant lessening of pathology severity even though the treatment was started only at the acute phase of infection (day 3 post-infection) and not immediately after animals were infected with *Chlamydia*. This observation, together with our results of significantly less shedding of Chlamydia in I-C-treated animals compared with placebo-treated groups, additionally support the hypothesis that the anti-chlamydial activity of I-C might be attributed to the prevention of the initial *C*. *trachomatis* contact with the epithelial host cells. Furthermore, this finding could also implicate that I-C per se might exert a positive local immunomodulatory effect, which might consecutively contribute toward the inflammation lessening and the infection resolution.

In conclusion, our findings show that the application of I-C reduces CtB infectivity in vitro and shedding of chlamydial EBs (GPIC) in vivo*.* Prolonged application of I-C might be needed for a significant improvement of the clinical picture. The reduction of shedding of infectious EBs is also of utter importance as it could contribute to a less effective transmission of chlamydial infection. Further studies, with the emphasis on the impact of I-C application schedule on the kinetics of a chlamydial infection are required to evaluate the full potential of I-C as a prophylactic and therapeutic treatment of ocular chlamydial infection.

## References

[CR1] Belij-Rammerstorfer S, Inic-Kanada A, Stojanovic M, Marinkovic E, Lukic I, Stein E, Montanaro J, Bintner N, Schurer N, Ghasemian E, Kundi M, Barisani-Asenbauer T (2016). Infectious dose and repeated infections are key factors influencing immune response characteristics in guinea pig ocular chlamydial infection. Microbes Infect.

[CR2] Bergsson G, Arnfinnsson J, Karlsson SM, Steingrimsson O, Thormar H (1998). In vitro inactivation of *Chlamydia trachomatis* by fatty acids and monoglycerides. Antimicrob Agents Chemother.

[CR3] Brown MA, Potroz MG, Teh SW, Cho NJ (2016). Natural products for the treatment of Chlamydiaceae infections. Microorganisms.

[CR4] Buck CB, Thompson CD, Roberts JN, Muller M, Lowy DR, Schiller JT (2006). Carrageenan is a potent inhibitor of papillomavirus infection. PLoS Pathog.

[CR5] Burillo CA, Fontenot JD, Phillips DM (1998). Sulfated polysaccharides block chlamydia infection in vitro, but do not protect mice from vaginal inoculation. Microb Pathog.

[CR6] Caldwell HD, Kromhout J, Schachter J (1981). Purification and partial characterization of the major outer membrane protein of *Chlamydia trachomatis*. Infect Immun.

[CR7] Carlucci MJ, Ciancia M, Matulewicz MC, Cerezo AS, Damonte EB (1999). Antiherpetic activity and mode of action of natural carrageenans of diverse structural types. Antivir Res.

[CR8] Chen JC, Stephens RS (1994). Trachoma and LGV biovars of *Chlamydia trachomatis* share the same glycosaminoglycan-dependent mechanism for infection of eukaryotic cells. Mol Microbiol.

[CR9] Coggins C, Blanchard K, Alvarez F, Brache V, Weisberg E, Kilmarx PH, Lacarra M, Massai R, Mishell D, Salvatierra A, Witwatwongwana P, Elias C, Ellertson C (2000). Preliminary safety and acceptability of a carrageenan gel for possible use as a vaginal microbicide. Sex Transm Infect.

[CR10] Cruz L, Meyers C (2013). Differential dependence on host cell glycosaminoglycans for infection of epithelial cells by high-risk HPV types. PLoS One.

[CR11] Daglia M (2012). Polyphenols as antimicrobial agents. Curr Opin Biotechnol.

[CR12] Damonte EB, Matulewicz MC, Cerezo AS (2004). Sulfated seaweed polysaccharides as antiviral agents. Curr Med Chem.

[CR13] Eccles R, Winther B, Johnston SL, Robinson P, Trampisch M, Koelsch S (2015). Efficacy and safety of iota-carrageenan nasal spray versus placebo in early treatment of the common cold in adults: the ICICC trial. Respir Res.

[CR14] ECDC (2015) STI trends in Europe: chlamydia rates stabilise while gonorrhoea numbers go up - See more at: http://ecdc.europa.eu/en/press/news/_layouts/forms/News_DispForm.aspx?ID=1285&List=8db7286c-fe2d-476c-9133-18ff4cb1b568#sthash.UTipMXVx.dpuf. http://ecdc.europa.eu/en/press/news/_layouts/forms/News_DispForm.aspx?ID=1285&List=8db7286c-fe2d-476c-9133-18ff4cb1b568. Accessed 05.02.2017 2016

[CR15] Fadel S, Eley A (2007). *Chlamydia trachomatis* OmcB protein is a surface-exposed glycosaminoglycan-dependent adhesin. J Med Microbiol.

[CR16] FDA SCOGS (Select Committee on GRAS Substances) (n.d.) http://www.accessdata.fda.gov/scripts/fdcc/?set=SCOGS

[CR17] Fernandez-Romero JA, Abraham CJ, Rodriguez A, Kizima L, Jean-Pierre N, Menon R, Begay O, Seidor S, Ford BE, Gil PI, Peters J, Katz D, Robbiani M, Zydowsky TM (2012). Zinc acetate/carrageenan gels exhibit potent activity in vivo against high-dose herpes simplex virus 2 vaginal and rectal challenge. Antimicrob Agents Chemother.

[CR18] Filipovic A, Ghasemian E, Inic-Kanada A, Lukic I, Stein E, Marinkovic E, Djokic R, Kosanovic D, Schuerer N, Chalabi H, Belij-Rammerstorfer S, Stojanovic M, Barisani-Asenbauer T (2017). The effect of infectious dose on humoral and cellular immune responses in *Chlamydophila caviae* primary ocular infection. PLoS One.

[CR19] Frick KD, Lietman TM, Holm SO, Jha HC, Chaudhary JS, Bhatta RC (2001). Cost-effectiveness of trachoma control measures: comparing targeted household treatment and mass treatment of children. Bull World Health Organ.

[CR20] Ghosh T, Pujol CA, Damonte EB, Sinha S, Ray B (2009). Sulfated xylomannans from the red seaweed Sebdenia polydactyla: structural features, chemical modification and antiviral activity. Antivir Chem Chemother.

[CR21] Gipson IK, Spurr-Michaud S, Argueso P, Tisdale A, Ng TF, Russo CL (2003). Mucin gene expression in immortalized human corneal-limbal and conjunctival epithelial cell lines. Invest Ophthalmol Vis Sci.

[CR22] Gonzalez ME, Alarcon B, Carrasco L (1987). Polysaccharides as antiviral agents: antiviral activity of carrageenan. Antimicrob Agents Chemother.

[CR23] Grassauer A, Weinmuellner R, Meier C, Pretsch A, Prieschl-Grassauer E, Unger H (2008). Iota-carrageenan is a potent inhibitor of rhinovirus infection. Virol J.

[CR24] Klimyte EM, Smith SE, Oreste P, Lembo D, Dutch RE (2016). Inhibition of human metapneumovirus binding to heparan sulfate blocks infection in human lung cells and airway tissues. J Virol.

[CR25] Lahaye M (2001). Developments on gelling algal galactans, their structure and physico-chemistry. J Appl Phycol.

[CR26] Leibbrandt A, Meier C, Konig-Schuster M, Weinmullner R, Kalthoff D, Pflugfelder B, Graf P, Frank-Gehrke B, Beer M, Fazekas T, Unger H, Prieschl-Grassauer E, Grassauer A (2010). Iota-carrageenan is a potent inhibitor of influenza A virus infection. PLoS One.

[CR27] Levendosky K, Mizenina O, Martinelli E, Jean-Pierre N, Kizima L, Rodriguez A, Kleinbeck K, Bonnaire T, Robbiani M, Zydowsky TM, O'Keefe BR, Fernandez-Romero JA (2015). Griffithsin and carrageenan combination to target herpes simplex virus 2 and human papillomavirus. Antimicrob Agents Chemother.

[CR28] Lietman T, Dawson C, Osaki S (1998). Ocular chlamydial infections. Int Ophthalmol Clin.

[CR29] Luo Z, Tian D, Zhou M, Xiao W, Zhang Y, Li M, Sui B, Wang W, Guan H, Chen H, Fu ZF, Zhao L (2015). Lambda-carrageenan P32 is a potent inhibitor of rabies virus infection. PLoS One.

[CR30] Mariotti SP, Pascolini D, Rose-Nussbaumer J (2009). Trachoma: global magnitude of a preventable cause of blindness. Br J Opthamol.

[CR31] McKim JM, Baas H, Rice GP, Willoughby JA, Weiner ML, Blakemore W (2016). Effects of carrageenan on cell permeability, cytotoxicity, and cytokine gene expression in human intestinal and hepatic cell lines. Food Chem Toxicol.

[CR32] Moelleken K, Hegemann JH (2008). The chlamydia outer membrane protein OmcB is required for adhesion and exhibits biovar-specific differences in glycosaminoglycan binding. Mol Microbiol.

[CR33] Molleken K, Schmidt E, Hegemann JH (2010). Members of the Pmp protein family of Chlamydia pneumoniae mediate adhesion to human cells via short repetitive peptide motifs. Mol Microbiol.

[CR34] Necas J, Bartosikova L (2013). Carrageenan: a review. Vet Mad.

[CR35] Norman J (2002). Epidemiology of female genital *Chlamydia trachomatis* infections. Best Pract Res Clin Obstet Gynaecol.

[CR36] Rahn C, Marti H, Frohns A, Frohns F, Blenn C, Leonard CA, Barisani-Asenbauer T, Stein E, Borel N (2016). Water-filtered infrared A reduces chlamydial infectivity *in vitro* without causing *ex vivo* eye damage in pig and mouse models. J Photochem Photobiol B.

[CR37] Rank R, Fox JG (2007). Chlamydial diseases. The mouse in biomedical research diseases.

[CR38] Rank RG, Dascher C, Bowlin AK, Bavoil PM (1995). Systemic immunization with Hsp60 alters the development of chlamydial ocular disease. Invest Ophthalmol Vis Sci.

[CR39] Rank RG, Whittum-Hudson JA (1994). Animal models for ocular infections. Methods Enzymol.

[CR40] Roberts JN, Buck CB, Thompson CD, Kines R, Bernardo M, Choyke PL, Lowy DR, Schiller JT (2007). Genital transmission of HPV in a mouse model is potentiated by nonoxynol-9 and inhibited by carrageenan. Nat Med.

[CR41] Schnitger K, Njau F, Wittkop U, Liese A, Kuipers JG, Thiel A, Morgan MA, Zeidler H, Wagner AD (2007). Staining of Chlamydia trachomatis elementary bodies: a suitable method for identifying infected human monocytes by flow cytometry. J Microbiol Methods.

[CR42] Stein E, Inic-Kanada A, Belij S, Montanaro J, Bintner N, Schlacher S, Mayr UB, Lubitz W, Stojanovic M, Najdenski H, Barisani-Asenbauer T (2013). In vitro and in vivo uptake study of Escherichia coli Nissle 1917 bacterial ghosts: cell-based delivery system to target ocular surface diseases. Invest Ophthalmol Vis Sci.

[CR43] Stephens RS, Koshiyama K, Lewis E, Kubo A (2001). Heparin-binding outer membrane protein of chlamydiae. Mol Microbiol.

[CR44] Talarico LB, Damonte EB (2016). Characterization of *in vitro* dengue virus resistance to carrageenan. J Med Virol.

[CR45] Talwar GP, Garg S, Dhar V, Chabra R, Ganju A, Upadhyay SN (1995). Praneem polyherbal cream and pessaries with dual properties of contraception and alleviation of genital infections. Curr Sci.

[CR46] Taraktchoglou M, Pacey AA, Turnbull JE, Eley A (2001). Infectivity of *Chlamydia trachomatis* serovar LGV but not E is dependent on host cell heparan sulfate. Infect Immun.

[CR47] Taylor HR, Burton MJ, Haddad D, West S, Wright H (2014). Trachoma. Lancet.

[CR48] Vera J, Castro J, Gonzalez A, Moenne A (2011). Seaweed polysaccharides and derived oligosaccharides stimulate defense responses and protection against pathogens in plants. Mar Drugs.

[CR49] WHO (2014). Alliance for the global elimination of blinding trachoma by the year 2020. Wkly Epidemiol Rec.

[CR50] Yamashita S (2001). In vitro bacteriostatic effects of dietary polysaccharides. Food Sci Technol Res.

[CR51] Yang B, Bhattacharyya S, Linhardt R, Tobacman J (2012). Exposure to common food additive carrageenan leads to reduced sulfatase activity and increase in sulfated glycosaminoglycans in human epithelial cells. Biochimie.

[CR52] Yasin B, Harwig SS, Lehrer RI, Wagar EA (1996). Susceptibility of *Chlamydia trachomatis* to protegrins and defensins. Infect Immun.

[CR53] Zaretzky FR, Pearce-Pratt R, Phillips DM (1995). Sulfated polyanions block *Chlamydia trachomatis* infection of cervix-derived human epithelia. Infect Immun.

[CR54] Zhang JP, Stephens RS (1992). Mechanism of *C*. *trachomatis* attachment to eukaryotic host cells. Cell.

